# Editorial: Effectiveness of exercise and diet on movement disorders

**DOI:** 10.3389/fnagi.2025.1571000

**Published:** 2025-02-20

**Authors:** Adérito Seixas, Cláudia Silva, Mario Bernardo-Filho, Redha Taiar, Danúbia da Cunha de Sá Caputo

**Affiliations:** ^1^FP-I3ID, FP-BHS, Escola Superior de Saúde Fernando Pessoa, Porto, Portugal; ^2^FP-I3ID, FP-BHS, Universidade Fernando Pessoa, Porto, Portugal; ^3^Laboratório de Vibrações Mecânicas e Práticas Integrativas, Departamento de Biofísica e Biometria, Instituto de Biologia Roberto Alcantara Gomes and Policlínica Universitária Piquet Carneiro, Universidade do Estado do Rio de Janeiro, Rio de Janeiro, Brazil; ^4^MATériaux et Ingénierie Mécanique (MATIM), Université de Reims Champagne-Ardenne, Reims, France

**Keywords:** exercise, physical activity, diet, movement disorders, disability, quality of life, functionality

## Introduction

Movement disorders (MD) are complex neurological conditions affecting the amount and fluidity of available movement and movement control. These conditions have multiple possible causes and do not fit into other established diagnostic categories. MD, which include conditions such as Parkinson's disease (PD), multiple system atrophy (MSA), progressive supranuclear palsy (PSP), corticobasal syndrome, cervical dystonia and others, have a significant impact on the quality of life of individuals worldwide but the prevalence and incidence rates vary by condition and geographical area (Abbas et al., 2018).

The prevalence of MD increase with age (Wenning et al., 2005), with essential tremor and PD being more common (Luo et al., 2025; Song et al., 2021). The spectrum of movement impairments is very broad, with bradykinesia or hypokinesia at one end of the spectrum and tremor, dystonia, chorea, stereotypies, athetosis, myoclonus and other dyskinesias at the other end of the spectrum (Jankovic and Lang, 2021). In hyperkinetic disorders, the abnormal involuntary movements may occur at rest or during voluntary movements and may be rhythmic (e.g., tremor), sustained (e.g., dystonia) or irregular (e.g., chorea and myoclonus) (Arabia et al., 2022; Novellino et al., 2009; Perju-Dumbrava and Kempster, 2020; Stephen et al., 2023). On the other hand, hypokinetic disorders are characterized by a reduction in voluntary movements and/or difficulty in initiating movements (Jankovic and Lang, 2021). MD often have no identifiable cause and are referred to as primary MD, as opposed to secondary MD, which have identifiable causes, such as infection (Desai et al., 2024; Divya et al., 2024). Both groups present with both motor and non-motor symptoms are present, and the diagnosis and management of these conditions is a challenging task for healthcare professionals, especially knowing that diagnostic criteria frameworks are diverse (Mestre et al., 2023).

For a complete assessment, historical—family and personal, including birth history and early developmental abnormalities—and clinical features obtained during the physical examination and laboratory testing are of paramount importance (Jankovic and Lang, 2021). In addition, technology—such as apps and wearables—has shown promising results in increasing the objectivity of the assessment (Caroppo et al., 2025; Vanmechelen et al., 2023; Willemse et al., 2024) but proper validation studies are needed to increase the confidence of clinicians in implementing these solutions in clinical practice.

The management of people with MD is also challenging, as they often experience difficulties with mobility, balance and activities of daily living, and non-motor symptoms, such as cognitive changes and psychiatric symptoms also manifest as the condition progresses. Given the progressive nature of these conditions, a palliative approach can help throughout the clinical course of the complex and long-term symptoms (Miyasaki et al., 2022). Pharmacological therapy is a cornerstone in the management of MD (Jankovic, 2009; Pirker et al., 2023) but other approaches, such as gene therapy (e.g. Kim and Chang, 2024), are becoming increasingly relevant. Moreover, increasing evidence highlights the therapeutic potential of non-pharmacological approaches such as exercise therapy, and nutrition-based interventions, to reduce the progression and severity of motor and non-motor symptoms (Ferrazzoli et al., 2022; Lister, 2020; Macías-García et al., 2024; Zhang et al., 2024).

The aim of this Research Topic was to share ideas, approaches, opinions, and comments on the latest research providing evidence on the mechanisms and safety of exercise- and diet-induced changes (e.g. neuroprotective, anti-inflammatory and immunological) and strategies to improve adherence and empowerment of people with MD in different contexts, as these are gaps that still need to be filled with high quality evidence.

This Research Topic includes eight papers that address these questions. Four of these are original research papers and four are literature reviews that deepen our understanding of these interventions and their practical applications. Among the original research contributions, Mateus and Castro Caldas reported on three cases of supranuclear palsy, describing the benefits of physiotherapy through exercise in improving functionality and quality of life; a cross-sectional study strengthened the knowledge on the association between motor function and health-related quality of life in people with PD (Ge et al.), providing insights on which aspects should be tackled by healthcare professionals; Yaqoob et al. have demonstrated that an accelerometer-based assessment of gait and sit-to-stand tasks in people with cervical dystonia has good agreement with direct observation; and Toloraia et al. presented a non-randomized study demonstrating the benefits of a multimodal (exercise, speech and cognitive) training programme in improving motor and cognitive function in people with PD.

The systematic reviews published in this Research Topic have provided valuable insights into the quality and the certainty of the existing evidence that can guide clinicians working with people with MD, as well as gaps that may guide future research in this area. Homann et al. showed that the existing randomized controlled trials analyzing the benefits of Vitamin D supplementation in later life show a potential benefit on a very limited number of MD. However, the very limited number of trials mainly led the authors to conclude that there is a clear need for further clinical research in this area. For exercise interventions, the evidence is more robust and three systematic reviews provide valuable insights for both clinicians and researchers. Yuan et al. presented a network meta-analysis identifying specific exercise modalities and dosages that significantly improve functionality in patients with PD, with aquatic exercise emerging as the most effective modality. These findings are partially challenged by Liu et al., who concluded that hydrotherapy had a positive maintenance effect on balance function in this population, but no significant long-term effects on motor function, mobility, and quality of life. Finally, Cui et al. highlighted the importance of adherence to the ACSM recommendations when prescribing exercise to patients with PD, as interventions with higher compliance to recommendations were more beneficial in improving motor function, balance, mobility, and quality of life in this population.

Taking into account the contributions to this Research Topic, the body of evidence on exercise and diet interventions in MD has been strengthened. However, there is less evidence on dietary interventions.

## Conclusion

As the prevalence of MD is expected to increase, research into the management of these conditions is needed. Diet and exercise interventions are key components of a multidisciplinary rehabilitation strategy for people with MD, but research is lacking, particularly on dietary interventions. This Research Topic contributes to the growing body of evidence supporting exercise and dietary interventions for MD. We hope that these findings will inspire further research and inform clinical practice to improve the quality of life of people affected by these challenging conditions.
